# Cost-effectiveness analysis of COVID-19 screening strategy under China's dynamic zero-case policy

**DOI:** 10.3389/fpubh.2023.1099116

**Published:** 2023-05-09

**Authors:** Haonan Li, Hui Zhang

**Affiliations:** ^1^School of Medical Business, Guangdong Pharmaceutical University, Guangzhou, Guangdong, China; ^2^Guangdong Health Economics and Health Promotion Research Center, Guangzhou, Guangdong, China

**Keywords:** COVID-19, cost-effectiveness, screening strategy, agent-based model, dynamic zero-case policy, China

## Abstract

This study aims to optimize the COVID-19 screening strategies under China's dynamic zero-case policy through cost-effectiveness analysis. A total of 9 screening strategies with different screening frequencies and combinations of detection methods were designed. A stochastic agent-based model was used to simulate the progress of the COVID-19 outbreak in scenario I (close contacts were promptly quarantined) and scenario II (close contacts were not promptly quarantined). The primary outcomes included the number of infections, number of close contacts, number of deaths, the duration of the epidemic, and duration of movement restriction. Net monetary benefit (NMB) and the incremental cost-benefit ratio were used to compare the cost-effectiveness of different screening strategies. The results indicated that under China's COVID-19 dynamic zero-case policy, high-frequency screening can help contain the spread of the epidemic, reduce the size and burden of the epidemic, and is cost-effective. Mass antigen testing is not cost-effective compared with mass nucleic acid testing in the same screening frequency. It would be more cost-effective to use AT as a supplemental screening tool when NAT capacity is insufficient or when outbreaks are spreading very rapidly.

## 1. Introduction

Coronavirus disease 2019 (COVID-19), is an infectious disease that causes fever, cough, shortness of breath, pneumonia, and lung infections. The pandemic poses a threat to the security of all humanity and has a huge negative effect on the economy, stability, and culture of countries around the world. A previous study estimated that the first wave of COVID-19 in China resulted in 2647 billion RMB losses (1). At present, China has effectively prevented large-scale outbreaks through the implementation of the strategy of “external prevention of importation and internal prevention of rebound” and the policy of “dynamic zero-case policy”(2). However, global COVID-19 is still in a pandemic state, Omicron is still sweeping across the world, and the sporadic cases and localized outbreaks in China are a reminder that the risk of large-scale outbreaks remains.

**Figure 1:**
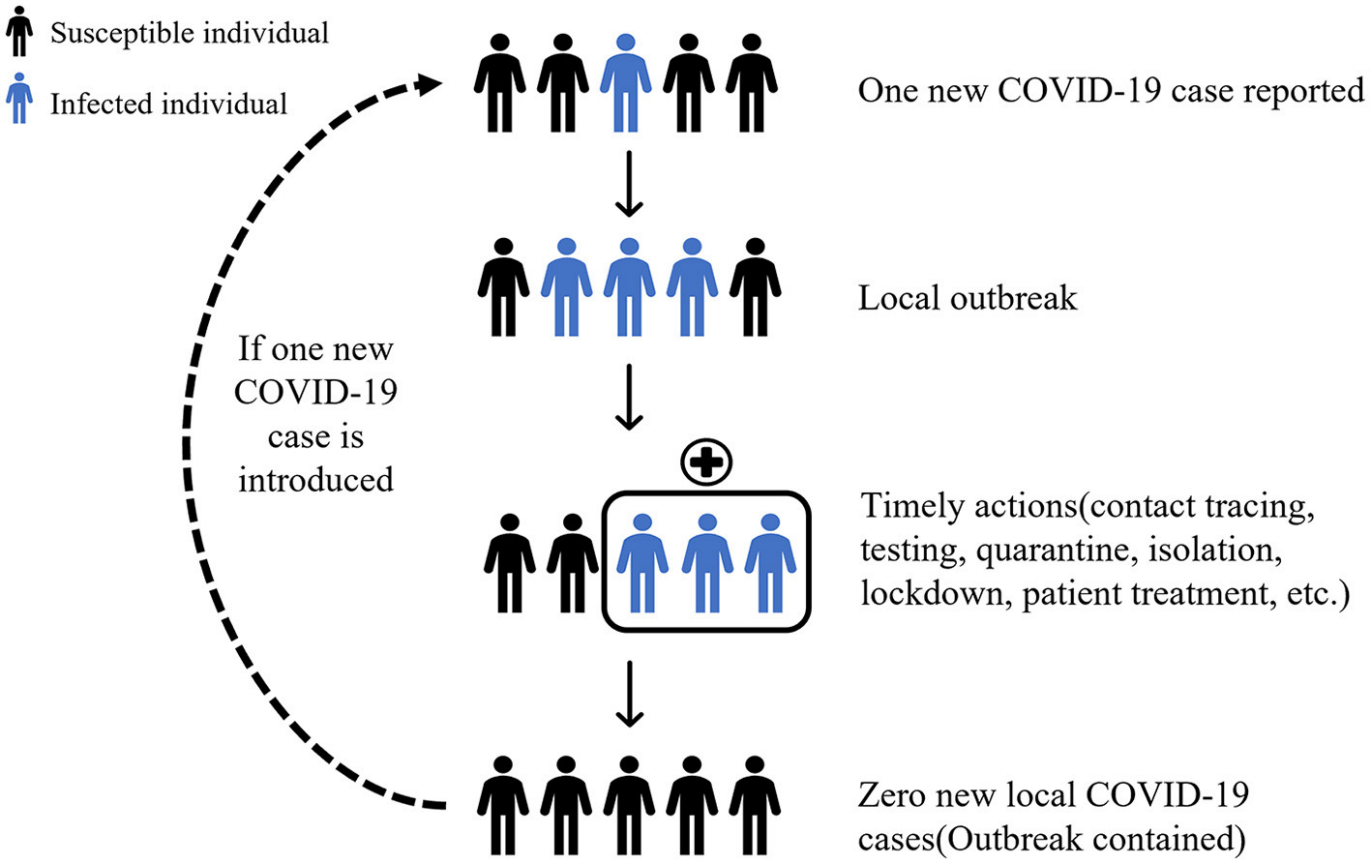
Schematic of dynamic zero-case policy.

Dynamic Zero-Case Policy is to contain domestic virus flare-ups through timely actions. Once a localized outbreak has occurred, rapid and accurate epidemiological investigations are carried out to identify the source of infection, which is then combined with regional mass screening, contact tracing, quarantine, isolation, and movement restriction to break the chain of transmission and contain the spread of the outbreak (Figure 1).

Mass screening helps to detect asymptomatic infections promptly and reduces community transmission (3). In addition, it can help decision-makers make more scientific judgments about ongoing community transmission and flexibly adjust prevention and control measures accordingly. Due to the different epidemiological situations and prevention and control policies, there is currently no standardized screening strategy for COVID-19 that fits all countries. Moreover, the cost-effectiveness of different COVID-19 screening strategies under China's dynamic zero-case policy has not been fully explored and demonstrated. Therefore, this study aimed to assess the cost-effectiveness of different screening strategies for COVID-19 in different scenarios to optimize screening strategies and reduce losses from localized outbreaks.

## 2. Methods

### 2.1. Comparator strategies

The most commonly used testing methods for COVID-19 are nucleic acid testing (NAT) and antigen testing (AT) in China. Based on the latest COVID-19 disease prevention and control guidelines (2), nine competing strategies consisting of different screening frequencies and testing methods were designed and compared in this study (Table 1). Considering the enormous challenges posed by the highly infectious Omicron variant for outbreak control, and to better simulate the real situation and more comprehensively assess different screening strategies, two scenarios were set for this study: (1) Scenario I: the outbreak spreads slowly, epidemiological investigations were carried out accurately, and close contacts can be traced and isolated promptly; (2) Scenario II: the outbreak develops rapidly, the transmission chain was difficult to sort out, and a proportion of close contacts cannot be traced and isolated promptly.

**Table 1:** COVID-19 community screening strategies.

**Screening strategies**	**Frequency of NAT**	**Additional AT**
S1	Once a day	–
S2	Once every 2 days	–
S3	Once every 3 days	–
S4	Once every 4 days	–
S5	Once every 5 days	–
S6	Once every 6 days	–
S7	Once every 7 days	–
S8	Once a day	Once a day (12 h from the NAT)
S9	Once every 2 days	Once every 2 days (24 h from the NAT)

### 2.2. Population and time Horizon

Community-based grid governance can be effective in helping to reduce or even stop outbreaks (4), hence the target population for this study was all community residents in China. All imported cases from abroad were excluded. The time horizon of the study was a localized outbreak, starting with the introduction of one infected case and ending with no un-isolated infected cases in the community.

### 2.3. Model

#### 2.3.1. Model summary

Mathematical models based on the SEIR framework have proven to be excellent tools for simulating and predicting the spread of infectious diseases and for evaluating prevention and control measures (5–7). However, these models do not capture individual differences, individual-to-individual, and individual-to-group effects, and are not flexible enough to fully assess different prevention and control measures. To address the above shortcomings, a stochastic agent-based model (ABM) was used to simulate the COVID-19 outbreak in this study. ABM is a method of simulating the behavior and interactions of autonomous agents in a particular environment across time steps (8). NetLogo software (Wilensky, Northwestern University) was used for modeling and running.

#### 2.3.2. Model description

**Figure 2:**
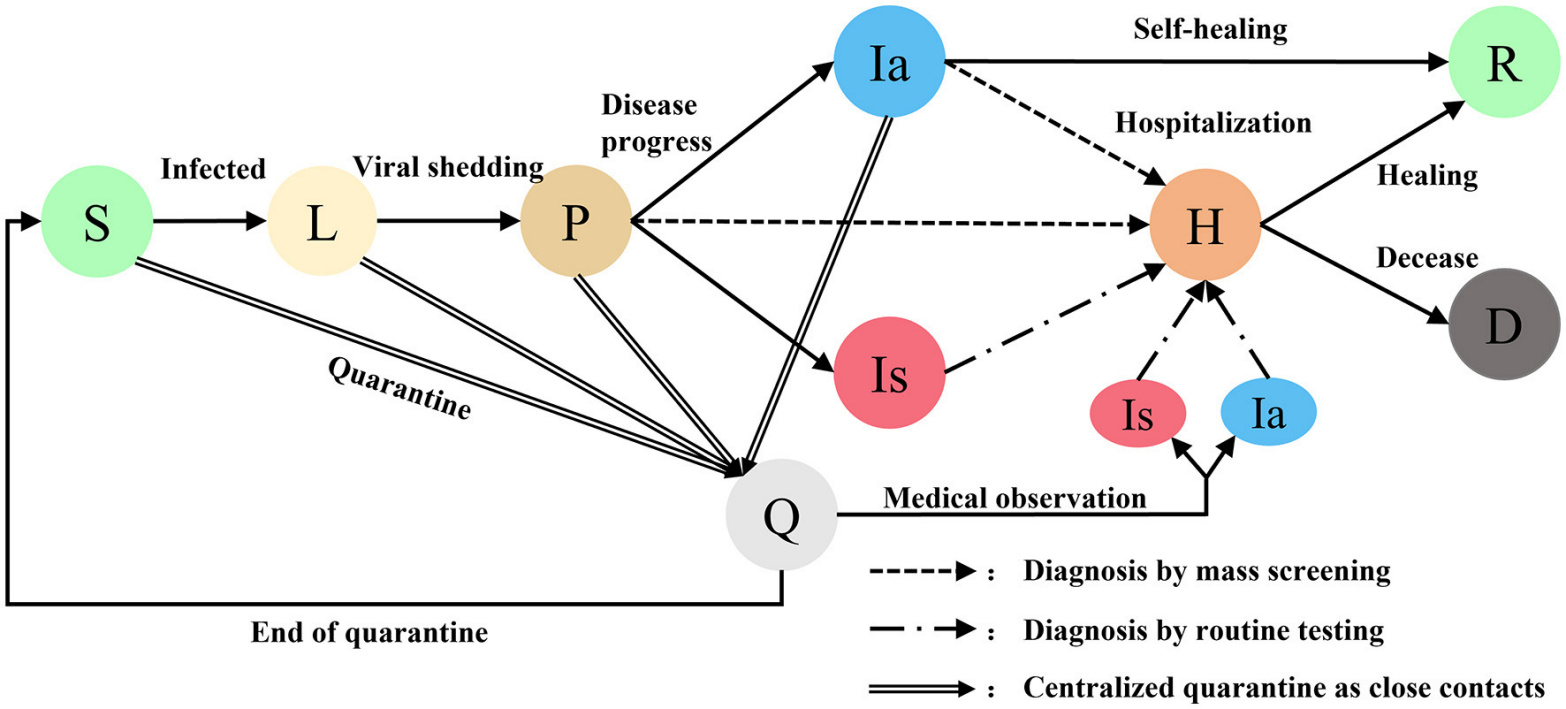
Different states of agents in COVID-19 transmission model. Susceptible S, Latent L, Pre-symptomatic P, Quarantined Q, Asymptomatic Ia, Symptomatic Is, Hospitalized H, Recovered R, Deceased D.

Agents are generated at random coordinates and move randomly within a virtual community. Initially, all agents are at the state of susceptible (S) except for a preset latent (L) infection. Latent infections become infectious and detectable after progressing to pre-symptomatic (P). Pre-symptomatic agent progress to asymptomatic infection (Ia) or symptomatic infection (Is) in a proportion. Symptomatic infections will be hospitalized (H) as confirmed cases after routine testing. When the number of confirmed cases is >0, regional mass screening and close contact tracking procedures will be initiated. Close contacts will be quarantined (Q) after being tracked. Asymptomatic infections are still infecting other agents unless they are hospitalized after diagnosing by mass screening or recovered (R) after self-healing. When the number of confirmed cases is >50, a community lockdown procedure will be initiated, all agents are not allowed to move or contact other agents. Hospitalized agents will progress to recovered or deceased (D). When there are no unquarantined infected persons in the community means that the outbreak is contained, all intervention procedures such as community lockdown will be stopped and the simulation will be aborted (Figure 2).

#### 2.3.3. Model parameters

Model parameters were mainly derived from previous studies, expert opinions, and fieldwork (Table 2). Population size was set to 6,000, which is approximately equal to the population of a small-scale community in China. Infectious capability of infections was based on the probability of infection in close contacts in previous studies (22) and adjusted for the basic regeneration number measured in this model. The time required for nucleic acid testing, including sampling, transfer, and laboratory testing, was based on expert opinion (9). Maximum mobility of agents and sizes of simulation space were adjusted for population density reasonably. Baseline values for epidemiological parameters were mainly taken from studies related to Omicron, as it is now the main prevalent strain worldwide (13–16, 18). In scenario I, maximum time needed for tracking close contacts was set to 72 h, and quarantined probability of close contact was set to 100%. In scenario II, these two values were set to 120 h and 75%.

**Table 2:** Value and source of model parameters.

**Parameter**	**Base-value**	**Source**
**Global Parameter**		
Sizes of simulation space (patches)	60*60	Assumption
Population size	6,000	Assumption
Time unit (hour/tick)	1	Assumption
Random seed number	1–1,000	–
Time required to obtain routine NAT results after sampling (hours)	4	Expert opinion (9)
Time required to obtain mass NAT results after sampling (hours)	8	Expert opinion (9)
Time required to obtain AT results after sampling (hours)	0.25	Expert opinion (9)
Sensitivity of NAT (%)	100	Assumption
Sensitivity of AT (%)	70	(10–12)
**Agent properties**		
Maximum mobility (patches/ticks)	0.5	Assumption
Infectious capability (%)	10	Assumption
**Epidemiological parameter**		
Asymptomatic infection rate (%)	35	(13)
Incubation period (days)	3	(14)
Infectious period for symptomatic infections (days)	7	(15)
Infectious period for asymptomatic infections (days)	4	(15)
Basic reproductive number	6.33	(16)
Infection fatality rate (%)	0.09	(17)
**Cost parameter**		
Proportion of mild/moderate, severe, and critical case of non-booster-vaccinated individuals (%)	81.5;13.8;4.7	(1, 7)
Proportion of mild/moderate, severe, and critical case of booster-vaccinated individuals (%)	96.7;3.3;0.0	(18)
Booster vaccination coverage (%)	71.7	Previous studies
Average medical cost for a confirmed case of non-severe, severe, critical (US$)	800; 7,513; 21,620	(1, 7)
Weighted average medical cost per case (US$)	1,501.28	Calculated
Single sample nucleic acid test cost (US$)	2.29	(19)
Pooled sample nucleic acid tests cost (US$)	0.43	(19)
Antigen test cost (US$)	0.86	(20)
Per capital GDP (US$)	11,568	(21)
National daily average salary (US$)	31.69	Calculated
Daily quarantine costs for close contacts (US$)	57.14	Fieldwork
**Other parameters**		
Working time lost of close contacts (days)	7	(2)
Working time lost of mild/moderate, severe, and critical case (days)	29.71; 33.92; 35.35	Calibrated (1, 7)
Weighted average working time lost per case (days)	30.05	Calculated
Lifetime working years lost for COVID-19 fatalities (years)	10.23	(1, 7)

Considering the huge impact of COVID-19 on productivity, societal perspective was adopted in this study. Both direct and indirect costs are included in the cost measurement. Indirect costs are calculated using the human capital approach (23). Cost parameters came from two studies of the disease burden of the first wave of COVID-19 in China (1, 7), and were calibrated according to the latest data and prevention and control policies (2). For example, the “14 + 14” quarantine policy was replaced by “7 + 3”. The centralized quarantine period for close contacts was shortened to 7 days, followed by 3 days of health monitoring, during which they could go to work with proper personal protective measures. Thus, working time lost of close contacts was calibrated to 7 days. The average medical costs and average work time lost per case were weighted by booster vaccination coverage and the proportion of clinical types. Due to the short time horizon of the study, costs were not discounted. All costs are converted to U.S. dollars (USD) at the exchange rate (1 USD = 7 RMB).

#### 2.3.4. Key assumptions

Limited by the accessibility of some data and for the sake of model streamlining, the key assumptions of this study are mainly as follows:

(1) All infectious individuals have the same infectious capacity, and the infectious capacity does not change over time.
(2) Patients assumed to be non-infectious and not at risk of a second infection.
(3) Infections cannot infect other individuals during isolation.
(4) Individuals receive a NAT on days 1, 2, 3, 5, and 7 during quarantine according to China's COVID-19 disease prevention and control guidance.
(5) The time required for NAT or AT is ignored.
(6) Confirmed cases will be promptly isolated and treated.
(7) Deaths not due to COVID-19 infection (Background mortality) were not simulated.

### 2.4. Cost-effectiveness analysis

The outcome indicators of the outbreak simulation in this study were mainly the cumulative number of infections, close contacts, quarantined persons, deaths, duration of the outbreak, and length of community lockdown. The economic evaluation indicators for competing strategies were screening cost, total cost, and net monetary benefit (NMB). Based on the outcome indicators, the total cost calculation formula in the formula are as follows:


$$\begin{array}{l} \text{Total cost = }Cost_{i}*AI+Cost_{cc}*ACC\text{+ }Cost_{d}*AD\\ \text{ + }Cost_{at}*TAT\text{+ }Cost_{pnat}*TPNAT\\ \text{ + }Cost_{snat}*TSNAT\text{+ }Cost_{cl}*LCL \end{array}$$

Cost_i_ is the weighted average cost per case and contains both direct and indirect costs. Direct costs were based on costs of cases of different disease severities obtained from previous studies, weighted first by booster vaccination coverage and then by the proportion of cases of different disease severities with or without booster vaccination. After weighting in the same way to obtain the weighted average working time lost per case, the indirect costs were obtained by multiplying by the national daily average salary. AI is accumulative infections. Cost_cc_ is the total cost of each close contact including direct and indirect costs. Direct costs include staff allowances, accommodation for quarantine, meals, and NAT, were obtained from fieldwork. Indirect costs were derived by multiplying the number of days of quarantine by the national daily average salary. ACC is accumulative close contacts. Cost_d_ is the total cost per death, obtained by multiplying lifetime working years lost for COVID-19 fatalities obtained from previous studies by per capital GDP, and only the labor loss due to death is considered here.AD is accumulative deaths. Cost_at_ is the cost per sample for AT. TAT is the total number of antigen tests. Cost_pnat_ is the cost per pooled sample NAT. TPNAT is the total number of pooled sample NATs. Cost_snat_ is the cost per single sample NAT. TSNAT is the total number of single sample NATs. Cost_cl_ is the cost of community lockdown per day. Due to the short time horizon of this study, only the labor loss due to the community lockdown is considered here. Its calculation formula is as follows:


$$\text{Cos}{\text{t}}_{\text{cl}}=\text{n}*\text{NDAS}$$

Where n is the total number of agents who have not been quarantined, hospitalized, or died at the end of the simulation. NDAS is national daily average salary per person. LCL is the length of community lockdown. The detailed cost parameters are shown in Table 2.

To reflect the uncertainty of the outbreak and to reduce randomness, 1,000 simulations were performed for each competing strategy in each scenario. The mean and standard deviation of outcome indicators were reported. A one-way sensitivity analysis was performed to test the robustness of the results and to analyze the impact of variations in parameter values.

## 3. Results

### 3.1. Simulation results

**Figure 3:**
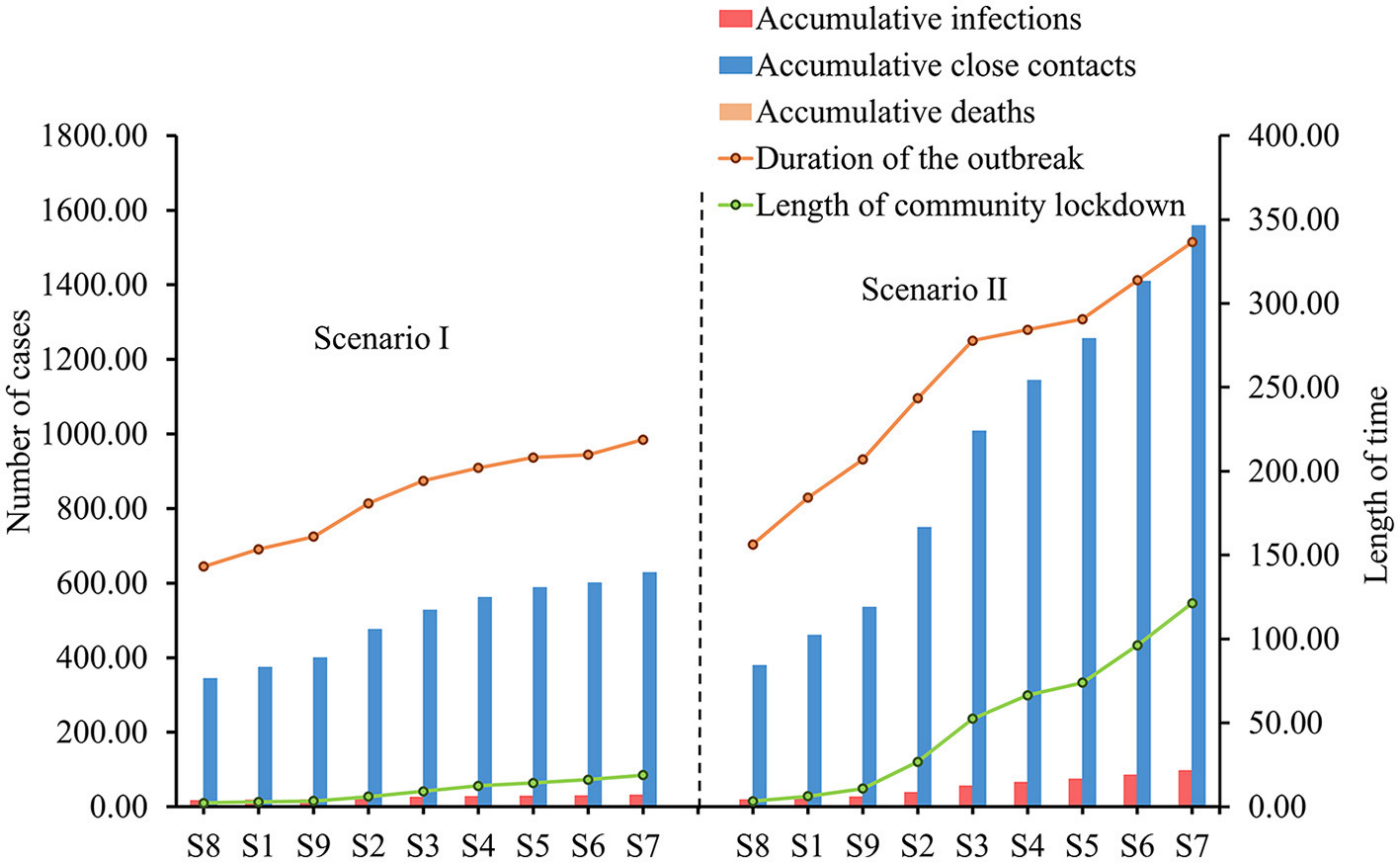
Simulation results of COVID-19 transmission under different strategies in two scenarios.

In Scenario I, the duration of the outbreak, accumulative infections, accumulative close contacts, and the length of the community lockdown trended flatly upward as the frequency of community screening decreased (Figure 3). This upward trend is more evident in scenario II, where the accumulative infections increased from 18.32 to 97.20, accumulative close contacts increased from 378.79 to 1558.75, the duration of the outbreak increased from 156.36 to 336.54 h, and the length of community lockdown increased from 3.37 to 121.38 h. Due to the low infection fatality rate and the small population size simulated, there were no deaths under all screening strategies (Table 3).

**Table 3:** COVID-19 transmission and cost effectiveness of different strategies.

**Strategy**	**Duration of the outbreak**	**Accumulative infections**	**Accumulative deaths**	**Accumulative close contacts**	**Length of community lockdown**	**Community screening cost (1,000 USD)**	**Total cost (1,000 USD)**	**Net monetary benefit (1,000 USD)**
**Scenario I**								
S1	153.48	17.95	0.00	374.11	2.89	9.83	275.79	297.45
S2	180.79	22.81	0.00	475.62	6.07	6.95	370.86	202.38
S3	194.25	25.52	0.00	527.19	9.27	5.48	431.04	142.20
S4	201.98	27.41	0.00	561.93	12.41	4.63	478.85	94.39
S5	208.15	28.75	0.00	587.50	14.25	4.09	510.33	62.91
S6	209.79	29.56	0.00	600.22	16.12	3.72	532.61	40.63
S7	218.78	31.10	0.00	628.46	18.87	3.51	573.24	-
S8	143.25	16.47	0.00	343.37	2.26	23.97	261.04	312.20
S9	160.99	19.17	0.00	399.27	3.48	15.43	304.15	269.09
**Scenario II**								
S1	184.17	22.36	0.00	460.11	6.26	13.13	283.38	1,409.32
S2	243.43	38.58	0.00	749.14	26.82	10.43	601.29	1,091.41
S3	277.87	55.87	0.00	1,007.94	52.48	8.67	930.70	762.01
S4	284.36	65.69	0.00	1,144.00	66.54	6.53	1,103.25	589.46
S5	290.63	74.30	0.00	1,256.22	73.96	5.90	1216.22	476.49
S6	313.77	85.58	0.00	1,408.91	96.28	5.64	1,452.28	240.43
S7	336.54	97.20	0.00	1,558.75	121.38	5.50	1,692.71	-
S8	156.36	18.32	0.00	378.79	3.37	28.66	226.16	1,466.54
S9	206.94	26.26	0.00	535.08	10.84	22.62	372.95	1,319.75

### 3.2. Cost-effectiveness results

**Figure 4:**
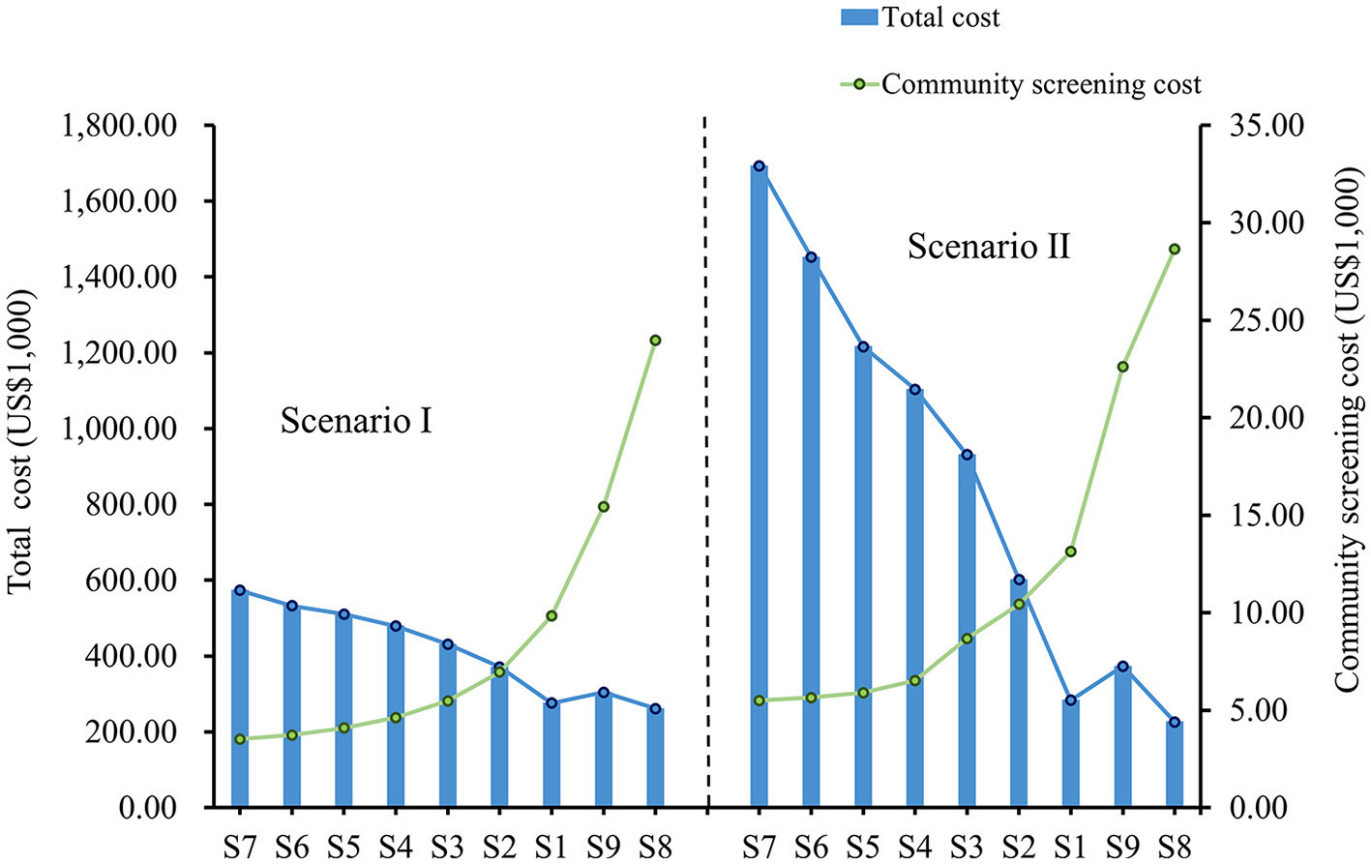
Total cost and community screening cost under different strategies in two scenarios.

In both scenarios, as the frequency of community screening increased, the cost of screening increased, but the total cost decreased, and this downward trend was more evident in scenario II (Figure 4). In both scenarios, S8 had the highest community screening cost but the lowest total cost, and S7 had the lowest community screening cost but the highest total cost. Compared to S7, S8 avoided a total economic loss of $312,204 in Scenario I and $1,466,543 in Scenario II. Although the screening frequency was the same for S1 and S9, S9 had a higher screening cost and a higher total cost in both scenarios. In addition, the total cost of S9 is lower compared to S2, which has a lower frequency of screening (Table 3).

### 3.3. Sensitivity analysis

**Figure 5:**
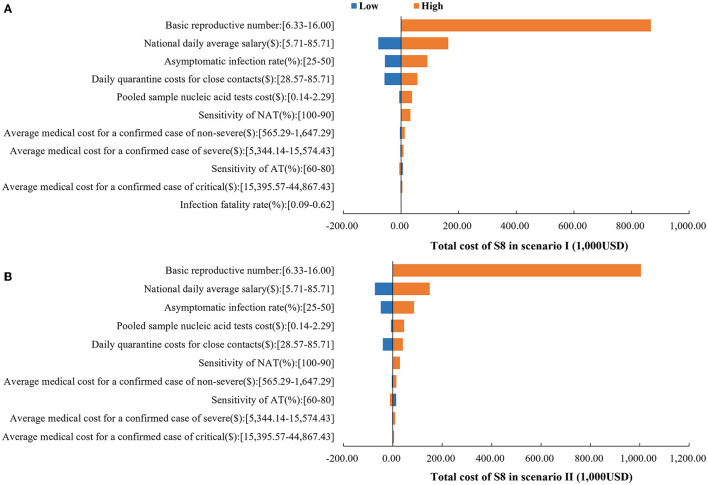
One-way sensitivity analysis results. **(A)** Impact of parameters on the total cost of S8 in scenario I. **(B)** Impact of parameters on the total cost of S8 in scenario II.

Detailed results of the sensitivity analyses are provided in Supplementary Tables 4, 5. Sensitivity analysis indicated that the base-case analysis was robust. The dominant strategy remained S8 when parameters were varied within the range. In both scenarios, the top 3 parameters that have the greatest impact on the total cost of S8 were basic reproductive number, national daily average salary and asymptomatic infection rate (Figure 5). Considering that S1 is the commonly used strategy in China during that period, we also provide the results of its one-way sensitivity analysis in Supplementary Tables 6, 7.

## 4. Discussion

This study evaluated different COVID-19 screening strategies under the current “dynamic zero-case policy” in China. The results showed that all indicators of outbreak size tended to decrease as the frequency of screening increased. This indicated that high frequency of screening could help contain the spread of the outbreak and reduce the size and burden of the outbreak, and this effect was more obvious in the case of a rapidly developing outbreak (Scenario II), which is consistent with the findings of previous studies (24–26). The most cost-effective screening strategy was daily NAT and an additional AT (S8). This suggested that such a high-frequency screening strategy is still cost-effective given the high transmissibility of the Omicron variant and the low cost of screening in China.

Meanwhile, the comparison of S1 with S9 revealed that mass AT is not cost-effective compared with mass NAT for the same screening frequency. This is probably because although AT has the advantage of convenience and speed, its sensitivity is lower compared to NAT, which may lead to missed and false detections, resulting in sequential transmission of outbreaks and increasing the total cost. Therefore, with the current low sensitivity of AT, it cannot completely replace NAT in community screening. However, additional AT in S9 would result in lower total costs compared to S2, which has a lower frequency of screening. The above suggested that using AT as a complementary screening tool would be more cost-effective in situations where NAT capacity is insufficient or the outbreak is spreading very rapidly.

It is worth noting that when an outbreak occurs, screening alone cannot block the spread of the outbreak. In this study, it was found during model simulation that if preventive and control measures such as close contact tracing and quarantine and restriction of population movement were removed, it would lead to all individual infections. Thus, the significance of mass screening is the timely detection of cases, and its cost-effectiveness is predicated on the combination of other prevention and control measures. This is also in line with previous studies (27–29).

Sensitivity analysis showed that among the cost parameters, the results were more sensitive to the national daily average salary. With the current low rate of severe illness and mortality, the economic burden of COVID-19 is mainly attributed to the loss of productivity. As found in other studies, productivity loss accounted for 99.8% of the total cost in the first wave of COVID-19 outbreak in China. Therefore, further research is needed on how to continuously optimize prevention and control measures, reduce the impact of the epidemic on productivity, and integrate epidemic prevention and control with economic development.

There are several limitations of this study. First, due to the short time horizon of this study, the health and cost impacts of long-COVID were not taken into account. Second, the simulated population size is relatively small, so the extrapolation of the model may be affected. Third, due to the lack of data, age, gender, and behavioral differences may lead to bias between the model and the real world. Fourth, a human capital approach was adopted in the measurement of indirect costs. And since some workers can choose to work from home, the indirect costs may be overestimated. Finally, the transmission capacity of infected individuals may vary with viral load, which was not taken into consideration in this study due to model streamlining.

Since the outbreak of COVID-19, China has been adjusting and optimizing its prevention and control strategy accordingly to the evolving situation. In January 2023, China's management of COVID was downgraded to the less strict class B from the current top-level class A, as the disease has become less virulent. From then on, China has entered a new phase in coordinating economic and social development and epidemic prevention and control.

Through a brief review we found that during the period when China adopted a “dynamic zero-case” strategy, although some cities strictly implemented the “dynamic zero-case” policy, there were cases where the size of the outbreak was larger than the simulated results of this study. The reasons for this phenomenon may be as follows: First, considering that epidemic control in China is community-based and the fact that too many samples may lead to excessively long model runs, this study is based on a simulation of 6,000 individuals in a single community in China, not on an entire city or an entire province. In the real world, provinces consist of many cities, and cities consist of many communities and individuals, and the increased sample size may result in a larger real-world epidemic size than in this study. The population density and geographic characteristics of different cities in the real world may also affect the spread of the epidemic. Second, in reality, although there have been large epidemics, there have also been many successful cases of “dynamic zero”. For example, the city of Shenzhen, with a population of 17.6 million and a highly mobile population, has implemented the “dynamic zero” policy well on more than one occasion, controlling the epidemic in a short period and keeping the prevalence rate at a low level. Third, some of the interventions in this model, such as close contact tracing and isolation, mass screening, and movement restrictions, are implemented according to set model parameter values. In contrast, in the real world, the effectiveness of interventions may be influenced by more factors such as affordability, civil compliance, country conditions, and government capacity, which may lead to higher data on the size of outbreaks in the real world than in this study. In light of the above, further research can be done in future studies to address the effects of affordability, civil compliance, national conditions, and government capacity on transmission in order to strengthen China's capacity for the prevention and control of infectious diseases, including COVID-19.

## 5. Conclusion

Under China's COVID-19 dynamic zero-case policy, high-frequency screening, such as daily NAT and an additional daily AT, can help contain the spread of the epidemic, reduce the size and burden of the epidemic, and is cost-effective. Mass AT is not cost-effective compared with mass NAT in the same screening frequency. It would be more cost-effective to use AT as a supplemental screening tool when NAT capacity is insufficient or when outbreaks are spreading very rapidly.

## Data availability statement

The original contributions presented in the study are included in the article/Supplementary material, further inquiries can be directed to the corresponding author.

## Author contributions

HL: study design, model design, parameters colleting, and data analyses. HZ: resources, supervision, and funding acquisition. HL and HZ: the original draft preparation and writing—review and editing. Both authors contributed to the article and approved the submitted version.
